# The “No bed syndrome” in Ghana — what, how and why? A literature, electronic and print media review

**DOI:** 10.3389/frhs.2023.1012014

**Published:** 2023-05-10

**Authors:** Linda Lucy Yevoo, Kezia Amerley Amarteyfio, Jewel Afriyie Ansah-Antwi, Lauren Wallace, Eunice Menka, Gifty Ofori-Ansah, Isaac Nyampong, Samuel Mayeden, Irene Akua Agyepong

**Affiliations:** ^1^ Research and Development Division, Dodowa Health Research Center, Ghana Health Service, Dodowa, Ghana; ^2^ Department of Surgery, Achimota Hospital, Ministry of Health, Accra, Ghana; ^3^ Department of Internal Medicine, Korle-Bu Teaching Hospital, Ministry of Health, Accra, Ghana; ^4^ Women, Media and Change, Accra, Ghana; ^5^ Greater Accra Region, Ningo Prampram District, Ghana Health Service, Accra, Ghana; ^6^ Alliance for Reproductive Health Rights, Accra, Ghana; ^7^ Policy, Planning, Monitoring and Evaluation Division, Ghana Health Service, Accra, Ghana; ^8^ Faculty of Public Health, Ghana College of Physicians and Surgeons (GCPS), Accra, Ghana

**Keywords:** emergency medical services, health system, Ghana, no bed syndrome, health priorities

## Abstract

**Objectives:**

“No bed syndrome” has become a familiar phrase in Ghana. Yet, there is very little in medical texts or the peer reviewed literature about it. This review aimed to document what the phrase means in the Ghanaian context, how and why it occurs, and potential solutions.

**Design:**

A qualitative desk review using a thematic synthesis of grey and published literature, print and electronic media content covering the period January 2014 to February 2021. Text was coded line by line to identify themes and sub-themes related to the research questions. Analysis was manual and with Microsoft Excel to sort themes.

**Setting:**

Ghana.

**Participants and Intervention:**

Not applicable.

**Results:**

“No bed syndrome” describes the turning away by hospitals and clinics of people seeking walk in or referral emergency care with the stated reasons “no bed available” or “all beds are full”. There are reported cases of people dying while going round multiple hospitals seeking help and being repeatedly turned away because there is “no bed”. The situation appears to be most acute in the highly urbanized and densely populated Greater Accra region. It is driven by a complex of factors related to context, health system functions, values, and priorities. The solutions that have been tried are fragmented rather than well-coordinated whole system reform.

**Discussions and recommendations:**

The “no bed syndrome” describes the challenge of a poorly functioning emergency health care system rather than just the absence of a bed on which to place an emergency case. Many low and middle income countries have similar challenges with their emergency health care systems and this analysis from Ghana is potentially valuable in attracting global attention and thinking about emergency health systems capacity and reform in low and middle income countries. The solution to the “no bed syndrome” in Ghana requires reform of Ghana's emergency healthcare system that takes a whole system and integrated approach. All the components of the health system such as human resource, information systems, financing, equipment tools and supplies, management and leadership need to be examined and addressed together alongside health system values such as accountability, equity or fairness in the formulation, implementation, continuous monitoring and evaluation of policies and programs for system reform to expand and strengthen emergency healthcare system capacity and responsiveness. Despite the temptation to fall back on them as low hanging fruit, piecemeal and ad-hoc solutions cannot solve the problem.

## Introduction

Responsiveness is one of the outcome goals of health systems. Responsiveness refers to the outcomes that can be achieved when institutions and institutional relationships in health systems are designed in such a way that they are aware of and respond appropriately to the universal and legitimate expectations of individuals. Responsiveness can be viewed from the angles of encouraging use of the system and safeguarding the right of users to safe and adequate care (1, 2). Responsiveness can also be viewed as a value within health systems (3).

Assured ready access and prompt attention for emergency medical, surgical and obstetric conditions is an important part of responsiveness within health systems. Emergency obstetric referral and care are a critical part of attaining the health-related Sustainable Development Goals (SDG) in Ghana as in much of Sub-Saharan Africa (4–6). Referral has been defined as “*a process in which a health worker at one level of the healthcare system, having insufficient resources (drugs, equipment, skills) to manage a clinical condition, seeks the assistance of a better or differently resourced facility at the same or higher level to assist in, or take over the management of, the client's case*” (7). Often patients presenting as emergencies have been referred. However, some also are brought in without a referral. Once a case is potentially life-threatening urgent attention should occur regardless of whether a person is brought to a clinic as an emergency with or without a referral.

Ghana developed a referral policy and guidelines in 2012 to help address some of the observed challenges in its referral systems. “The policy covers all types of referrals whether between different facilities or from one department of the same facility to another: and from primary through to tertiary care. It emphasizes the need to avoid delays in access to critical and emergency referral care, rights of referred patients to care and proper documentation of referrals. All public and private health facilities are expected to adhere to the policy and develop operational institutional guidelines to facilitate implementation” (8). Despite the comprehensive referral policy and guidelines Ghana's emergency referral system has been observed to be plagued by many shortcomings (9–12). These affect delivery of quality emergency services to patients, including pregnant women (13, 14). Several factors related to patients, health system and organizational challenges such as inadequate human and material resources, infrastructure, and context have been suggested to account in part at least for this problem. A recurring theme in the media and public discussions of the challenge of providing prompt and timely emergency care is a phenomenon referred to as the “no bed syndrome”. It is cited as contributing to Ghana's relatively high levels of maternal and neonatal mortality particularly in the Greater Accra Region (15, 16). In 2018, the death of a 70-year-old man after being turned away by multiple hospitals made media headlines and brought this issue which had already existed for a few years into acute public attention and triggered a parliamentary enquiry (17, 18).

What exactly is the phenomenon that is labelled as “no bed syndrome” in Ghana? Any resolution to the problem requires a clear understanding of the phenomenon, how and why it occurs and potential as well as actual solutions already tried. To inform ongoing policy discussions and the search for effective solutions a desk review was conducted with the review questions: “what does the term “no bed syndrome” refer to?”; “How and why does the “no bed syndrome” occur”; and “what interventions are in place or have been tried in Ghana to deal with the problem”? Reflecting the nature of the questions being asked the review team was multi-disciplinary with expertise in medical anthropology, clinical care, health policy and systems, communication, and media. The team also had a mix of early, mid-career and senior researchers and practitioners, reflecting the capacity building and use of evidence to inform policy and practice objectives of the project of which this work was a part.

## Materials, subjects and methods

The study design was a qualitative desk review using a thematic synthesis approach. Thematic synthesis is an approach to qualitative synthesis that involves coding of text, development of descriptive themes and a synthesis of analytical themes out of these to answer specific questions (19). We reviewed grey and published literature including dissertations, presentations, policies, legislation, administrative reports, and press statements as well as electronic and print media reports covering the period January 2014 (just before the onset of the SDG) to February 2021.

Key words and phrases used in all the searches were: “no bed syndrome”, “no bed”, “emergency care” “access”, “Ghana”, “Sub-Saharan Africa”, “Low- and middle-income countries”. We searched Google, Google scholar and PubMed. For student dissertations we searched the websites of the two oldest universities in Ghana with well-established Schools of Public Health, the University of Ghana and the Kwame Nkrumah University of Science and Technology. We also searched the websites of the Ghana Health Service and the Ministry of Health. The electronic and print media search was done using the Dow Jones Factiva software in March 2021. In Factiva we searched electronic and print media content from six media houses, three public (Graphic Online, Ghanaian Times and the Ghana News Agency) and three private (Daily Guide Network, The Ghanaian Chronicle, and MyJoyonline); and from the Ghana web. We purposively selected these as the most widely used public and private media outlets in Ghana.

Though the phrase “no bed syndrome” has been in widespread use by the media, public, non-medical and medical professionals, legislators, and policy makers in Ghana for several years now our search in the international peer reviewed literature for that phrase alone was not productive for the period of our review. Even as at the time of writing this paper a repeat search on 2 April 2022 in PubMed for the phrase “no bed syndrome” yielded the results “Quoted Phrase not found: No bed syndrome”.

The Google search, grey literature, print and electronic media searches were the main sources of information for answering most of our study questions. This review therefore relies on information from the electronic and print media where the term “no bed syndrome” is used to understand what the terminology refers to and why it is of policy and implementation relevance.

The inclusion criteria were all scholarly articles, grey literature such as press statement, policy briefs and dialogues, media articles within the six newspapers, that contained the search words and within the Ghanaian context. Exclusion criteria were documents that did not write about the no bed syndrome, problems with bed availability in hospitals and clinics, emergency referral access and/or emergency obstetric referral and care in Ghana.

Where abstracts were available, they were first reviewed to decide if the full text merited review. Where there were no abstracts, the full text was reviewed.

The Factiva search yielded 70 print and electronic media records for the search term “no bed syndrome”. The Google search initially turned up 16,400 results which was unmanageable. The search was refined using the search term combination “no bed syndrome” AND “emergency care access” AND “Ghana”. This yielded 37 records all of which were reviewed. Eight records were not relevant to the study based on the exclusion and inclusion criteria and were excluded. The remaining twenty-nine (29) articles, in addition to the seventy (70) media reports were fully reviewed and inform our analysis.

The selected articles were read thoroughly looking for themes and sub-themes related to “no bed syndrome”, emergency care and referral including emergency obstetric referrals. The coding of themes and sub-themes was done manually and then further analysed with text sorting of themes and sub-themes in Microsoft Excel. All analysis was done by the same three team members and reviewed by the others. It was not relevant to appraise quality of studies given the information available on our study questions was mainly from journalistic articles and descriptive studies. Our study questions were moreover about understanding framing and concepts.

We used the enhancing transparency in reporting the synthesis of qualitative research (ENTREQ) guidelines (20) to inform our reporting of results.

## Results

### What is the “no bed syndrome”?

The Ghanaian phraseology “No bed syndrome” is consistently used to describe a situation in which patients presenting at hospitals and clinics as emergencies are turned away, or asked to move on to another facility without any preliminary examination or management with the reason “no bed available”. Such patients may have been referred from one facility to another as a medical, surgical or obstetric emergency, or been brought directly to a hospital outpatients or emergency room. They may also be brought from the community without a referral in what is perceived to be a medical, surgical or obstetric emergency situation (10, 15, 16, 21, 22).

*“ “No bed syndrome” means patients who need emergency care, go through several facilities in Accra seeking medical attention but do not receive care on account of no bed, and eventually leads to the patient's death”* (23).

Its occurrence including in emergency obstetric care is most mentioned in the Greater Accra Region (24).

*“It is reported that the region is facing what they call “no bed syndrome”, where pregnant women who have referred are moved around from one facility to the other, being rejected on account of no bed. This situation results in the clients finally being attended to when the condition is very critical* (15).

Beyond the commonly stated reason when emergency patients are turned away because there is literally no bed available to place the patient on, the phraseology appears to cover underlying other issues often not stated when the patient is turned away. They are issues associated with long standing health system challenges and deficiencies in the state of the emergency healthcare system and the impact on care and outcomes (4, 21, 25–29). The “no bed syndrome” is therefore not just about physical non availability of a hospital bed to place a patient on, even if that is one of the most visible manifestations of inadequate emergency care access and responsiveness it describes. It is rather a complex issue that involves the turning away of clients in urgent need of health care sometimes at multiple points of care which relates to multiple failures and inadequacies of the emergency health care and referral system capacity and functioning.

### How and why does the “no bed syndrome” occur?

**Figure 1:**
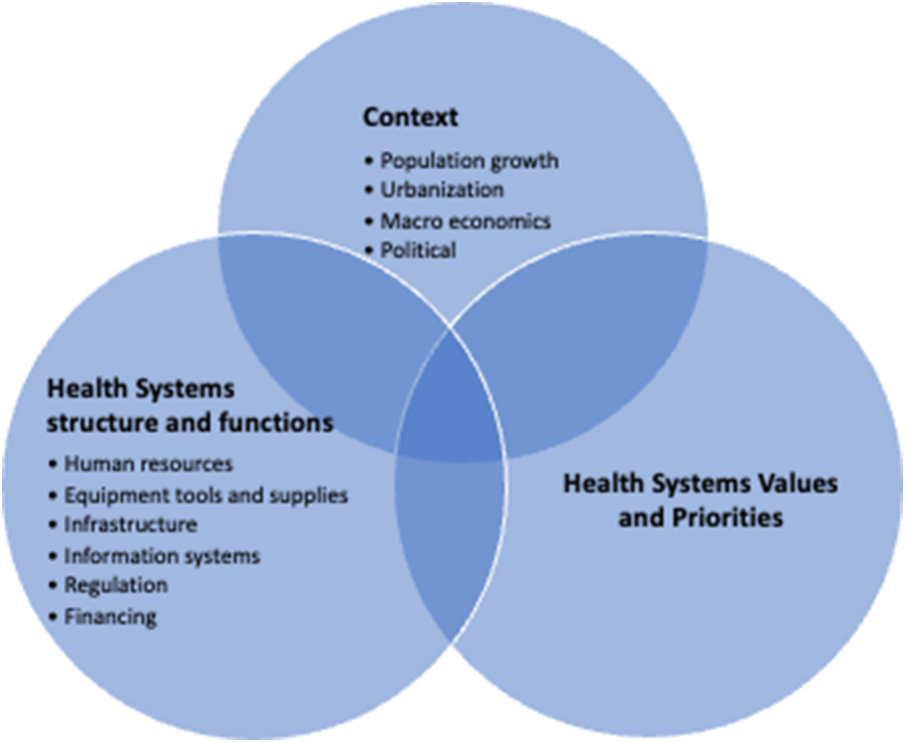
How and why does the “no bed syndrome” occur in Ghana?

To understand how and why the “no bed syndrome” occurs is to take a closer look at Ghana's emergency health care system. Figure 1 summarizes the review findings as to how and why the “no bed syndrome” occurs. They can be clustered into three broad categories of factors related to context, health system functions or building blocks, health system values such as equity and responsiveness; and health system priorities such as resource allocation and distribution. Rather than any one factor, the combination of contextual challenges, malfunctioning in aspects of the health system needed to support effective emergency referral systems and care, values and priorities drives the “no bed syndrome” and the related potentially avoidable morbidity, mortality and poor emergency health system responsiveness.

### Context

Contextual factors influencing the occurrence of the no bed syndrome are related to macro-economic, population, urbanization, and political factors. Ghana's population has grown rapidly in the decades since independence in 1957. Along with population growth has been urbanization (30) and an increasing dual burden of communicable and non-communicable diseases (31). Patients with complications of chronic non communicable conditions such as heart diseases, hypertension and diabetes sometimes have longer hospital stays, increasing bed occupancy rates in an already under-served system. The supply of human resource, infrastructure, equipment, tools, and supplies in the health system has not kept pace with these increases in demand and changes in disease burden. These interrelated factors combine to create a situation of overcrowding and congestion, especially in more densely populated urban areas. This is especially evident in public facilities, which are cheaper and therefore used by more people, with the number of patients outstripping bed and other resource capacities as both emergency and non-emergency cases compete for the limited resources available and have to be attended to in the same space and resource envelope (32). It hampers the ability to provide timely and responsive quality care (33). It also forces frontline health care providers, who in addition to the lack of resources to care for the patient may themselves be exhausted, stressed and frustrated (34), to adopt practices such as turning away clients with the explanation of “no-bed”, as part of coping behaviour.

On the macro-economic front, Ghana's economic growth for many years post-independence was slow, even stagnating or having some downturns in the seventies and eighties and has only picked up in recent years (35). This has limited the availability of government resources to expand capacity.

Political decisions to start and then abandon infrastructure projects related to changes in government emerged in our review as perceived contributors to the inadequate emergency care infrastructure that is one of the drivers of the “no bed syndrome” (24, 36–38). An illustrative example from periodic media reports on this theme is as below:

*“The KATH maternity ward project was started in 1974 by the Kutu Acheampong regime but the project has been abandoned by successive governments, resulting in deaths of pregnant women and babies at the facility”* (37).

### Health system priorities and values

Inadequate prioritization and related underfunding and relative neglect of assuring well distributed and equipped emergency health services by successive governments contributes to the “no bed syndrome”. The Accra Sports Stadium disaster on May 9, 2001, prompted the establishment of a national ambulance service after the findings and recommendation of the official commission of enquiry (38) drew attention to the role the lack of first aid facilities and emergency transportation systems played in the high death toll. Unfortunately, the strengthening of emergency care services appears not to have been carried much beyond the establishment of the national ambulance service (39, 40). As well as the non-existence of a national ambulance services until the early 2000s (9) functional emergency departments were a challenge. Even where emergency departments were set up, they were sometimes below international standards^1^. Emergency departments were observed to be often left out in the architectural designs of public primary healthcare facilities (24).

Historical and continuing contemporary decisions and prioritization of where to locate facilities may also be contributing to the problem in the densely populated and highly urbanized Greater Accra region where the problem is most acute and visible. The majority of the major referral hospitals such as the Korle-Bu teaching hospital (KBTH), Greater Accra Regional Hospital, 37 Military hospital and the Police hospital, are located in the city centre. The periphery that used to be rural is increasingly almost as urbanised as the centre with health and other infrastructure and services struggling to keep pace with population growth. It is therefore underserved (10).

### Health systems functions or building blocks

Interrelated to and working synergistically with the contextual factors are several health system functions or building blocks, related to financing, regulation, information systems and technology, human resources capacity, numbers and distribution, availability of equipment, tools, supplies, infrastructure, and emergency transportation.

### Financing

Resource constraints and under funding of the health system generally and emergency care specifically are a longstanding problem (36, 41). Inadequate procurement of tools, equipment and supplies required for emergency health service provision appeared in several media reports on the “no bed syndrome” e.g.

*“Government's refusal to increase bed numbers in numerous government hospitals has also propounded the issue of ‘no bed syndrome’ both in general medical emergencies and emergency obstetric care”* (42).

These problems were also reported in the media as further compounded by the National Health Insurance Scheme (NHIS) chronic indebtedness to health care facilities for services rendered to clients (29). Provider financial constraints sometimes made it difficult for facilities to restock needed emergency care resources such as medicines and devices. Sometimes when frontline providers turned patients away because of “no bed” it was also because of the of the lack of these other essentials that enable care rather than just the absence of a bed to place the patient on (see footnote 1).

### Regulation

Challenges related to various health regulatory bodies in the country such as the Health Facility Regulatory Authority (HeFRA) and Professional Councils such as the Medical and Dental Council (MDC) that are responsible for accreditation, monitoring and supervision towards assuring high quality standards emerged from the review as health system factors contributing to the “no bed syndrome” (see footnote 1). Sometimes the regulatory bodies, also under funded like the rest of the health sector, were unable to fully enforce the regulatory measures required by law. Health care facilities sometimes did not meet required standards or breached emergency referral protocols or process, citing reasons such as scarcity of both human and material resources. The reality of the scarcity of human and other resources sometimes created dilemmas for the regulatory authorities. Without some bending of rules and compromise in a resource constrained system the alternative might be no service at all.

### Information systems and technology

Health Management information systems and information technology also emerged as important health system factors through their effect on poor documentation and coordination and communication of referrals between facilities and between emergency transportation and facilities. Inadequate incorporation of Information Technology (IT) innovations in emergency service was cited as contributing to the “no bed syndrome” (41). It appeared a normal practice in emergencies where patients needed to be referred for the attending health providers to call the receiving facility using their personal mobile phone or directing the call for help informally to a friend at the next referral level. This was because of the absence of well-structured systems, such as a call centre that could coordinate, support, and facilitate timely communications for referral in emergencies (8). Other times, patients were just referred to the next level of care without any prior communication with the receiving facility to confirm their ability and readiness to take the case (43).

### Human resources for health

Inadequate health staff numbers, skills and distribution also contributed to the “no bed syndrome”. Emergency health care usually requires the efforts of skilled multi-professional teams with ready availability of staff such as anaesthetists, critical care physicians and nurses, surgeons, obstetricians, theatre nurses, and laboratory personnel among others. The very few skilled emergency and critical care staff tended to be concentrated in the teaching and bigger public hospitals located centrally, where they provided care as well as training to medical, nursing and other health care professionals (44) (See footnote 1). Frontline health workers in peripheral facilities multitasked as providers of both emergency and non-emergency care even though they might have little or no training skills and experience for emergency care, given the alterative was often “no care at all” (45). Emergency Medicine, surgery, nursing, and related specialty training opportunities for Ghanaian health professionals are limited (9). Training in emergency medicine and continuous professional development programmes in basic and advanced life support are only now emerging and the supply still needs to develop to meet the demand (9, 39, 46, 47).

Poor motivation and unprofessional attitudes were also reported as driving the “no bed syndrome” in the review (11). These appeared to be often driven by the context and systems. Practices such as poor adherence to emergency care guidelines, sending patients away without trying to triage or provide any kind of first aid were sometimes related to lacking the requisite skills and confidence to manage clients, feeling overwhelmed and worn-out from high workloads. Essential health staff absenting themselves from work, requests for upfront payment for emergency services and staff being disgruntled that they receive few incentives despite their heavy workloads were mentioned as contributors to the “no bed syndrome” (33, 44, 48, 52).

Inadequate monitoring and supervision of frontline providers by management staff were felt by some to be a factor fuelling what were seen as negative attitudes such as turning away emergencies (49). Facility managers on the other hand, sometimes struggled to know how to deal decisively and effectively with the given contextual challenges and somewhat narrow decision-making space. In the public sector human resource management including recruitment and salaries as well as budgets for capital investments such as infrastructure and major equipment are generally centralized. Facility managers are limited in what disciplinary action can be taken against staff indiscipline and may have limited say on priorities for infrastructure development or major equipment purchase. Management sometimes turned a blind eye to problems for fear they would lose critical staff for whom it was not clear where they would find a replacement (50).

### Equipment, tools and supplies including emergency transport

Inadequate supplies of emergency drugs, tools, logistics (including beds), laboratories, equipment, infrastructure, and transport emerged frequently as a contributor to the “no bed syndrome”. This included challenges with maintenance of broken-down emergency diagnostic equipment, or procurement of sub-standard quality (33, 43, 44, 51–55). Limited bed availability to meet the increases in patient numbers often resulted in healthcare workers in densely populated urban areas with already overcrowded public sector emergency rooms providing emergency care to patients on floors, benches, and chairs improvised as beds or referring patients to other facilities even when the health professionals with needed expertise to provide care were present in the referring facility (57).

In terms of emergency transportation, though the establishment of a national ambulance service after the 2001 stadium disaster was a major gain, the service remains under-funded and under-equipped in relation to the needs in the country (9). To fill in the gaps, people continue to use other means of transport services such as taxis and private cars to get emergency cases to hospitals and clinics. Sometimes even when available ambulances are challenged by the availability of patient trolleys and other essential ambulance equipment as well as inadequate numbers of trained technicians needed to give first aid and keep patients stable until they got to the referral centre.

In the highly urbanized and densely populated Greater Accra region of Ghana where the “no bed syndrome” has been and remains most visible, there are a multiplicity of private hospitals and clinics. In theory they could help relieve some of the strain in the public sector. However, like the public sector they also appeared to be challenged by inadequately trained human resources, as well as a lack of equipment, tools, and infrastructure for emergency care. It also appeared that concerns about their public image and reputation under these circumstances generated an unwillingness to take risk and a tendency to quickly refer patients with life threatening conditions to the public sector.

### What are the existing and potential solutions?

Our review identified several efforts to address directly or indirectly the “no bed syndrome” that have occurred over time in Ghana. Most of these efforts have been done in as fragmented single interventions and piecemeal approaches rather than based on a holistic assessment and a nationally coordinated and well synergized intervention. The efforts we identified from our review were mainly supply-side interventions, namely: administrative directives, health worker training, call centre and teleconsultation-based interventions, and donation of logistics, supplies and emergency transportation, new infrastructure, and software to monitor bed availability and referrals. Demand-side interventions such as rights-based approaches involving seeking legal redress appear to be uncommon (56).

### Administrative directives

Following the highly publicized death of a 70-year-old man after being turned away from several hospitals, the Ministry of Health (MOH) and Ghana Health Service (GHS) issued a directive to all public hospitals not to turn away any emergency cases regardless of their bed state. Rather, health facilities should rather aim at stabilizing patients that arrive in their facilities as emergencies and after which arrange for proper transfer to a higher referral hospital for care as needed (44, 63, 65). Public sector health facilities tried to adhere. The challenges that confront the emergency healthcare system such as inadequate numbers, skills and distribution of staff, infrastructure, equipment, tools and supplies were not immediately addressed and it is not clear how effective this directive has been in terms of saving lives since there was no evaluation available.

### Health worker training and deployment

In addition, in the wake of the “No Bed Syndrome”, the Ghana Health Service initiated training for medical doctors in basic life support and lifesaving skills under the continuous professional development programme of the Medical and Dental Council. We did not find any evaluation of impact.

One media report mentioned a Medical Outreach and re-deployment programme of doctors from the Korle Bu Teaching Hospital (KBTH) in the Greater Accra Region being deployed to some health facilities to help address the “no bed syndrome” (58).

### Call centre and teleconsultation-based interventions

In the Greater Accra region, pilot research has been conducted to establish and evaluate a call centre to coordinate referring and receiving referral facilities and provide up to date information on bed availability. It was targeted at obstetric and new-born referrals but was used for referrals for almost all cases. In the pilot, facilities referring women would call the centre and be guided as to which facilities had the needed expertise and available space to receive the client. The national ambulance service was also alerted to assist with the transportation of emergencies (15). An assessment of the call centre functioning noted the initiative had to a large extent had helped to minimise the effect of “no bed syndrome” on pregnant women and other emergency cases. However, the problems of poor funding and inadequate staffing were among the problems confronting the call centre (59, 60).

In the Amansie West district a teleconsultation program has been piloted that provides decision making support to community health workers for advise on the treatment of their patients, as well as help to manage emergency cases that are beyond their capacity and avoid unnecessary referrals (61). Calls have been made for more or scaled-up versions of these kinds of interventions e.g., (48). Like the other interventions, evaluation of impact on the ultimate outcomes of morbidity and mortality of medical surgical and obstetric emergencies of the teleconsultation-based interventions is currently not available. None of them have been applied at scale across the country.

Some facilities in the country such as Komfo Anokye Teaching Hospital (KATH), Social Security National Insurance Trust Hospital and Legon Hospital resorted to the use of technology to assist them in monitoring and assessing bed availability and referrals. For example, Korle-bu teaching hospital was trialling a pilot project on using a software that allows departments to keep track of beds in the hospital.

### Logistics, supplies, infrastructure and emergency transport provision

Governments, individuals, and corporate organisations have periodically donated beds, ambulances, mattresses etc., to different hospitals in various parts of the country (44, 62, 63). For example, the president of the country presented ten thousand hospital beds, under the infrastructure for Poverty Eradication Programme (IPEP), to be distributed to hospitals across the country (64). Sometimes new infrastructure has been commissioned (65). In addition, ambulances were also procured for the National Ambulance Service to increase their fleet numbers, as well as replace ambulances that had broken down. The recurrent costs of running and maintaining the emergency transport system appear to be a challenge, and we found reports of the emergency transportation services needing to demand for money for fuel for example, before patients were transported by ambulances (9).

## Discussion

The “no bed syndrome” is popular phraseology used in Ghana to describe the turning away of emergency cases from public and private hospital and clinic outpatient's department (OPD) and emergency rooms with the stated reason “no bed available”. The syndrome is the manifestation of an inadequate emergency health care system. What is known as the “no bed syndrome” in Ghana is effectively a synonym for inadequate emergency health care access. The problem is especially acute in densely populated highly urbanized regions like Greater Accra, where the rapid growth in population from a combination of migration and natural increase has resulted in demand outstripping supply of emergency care infrastructure in a system that was already under-funded and under-capacitated.

Inadequate emergency health care access challenges similar to what we have found in this review are documented from other countries in Sub-Saharan Africa (SSA). For example, a review of key barriers to the provision of emergency and surgical care in Ghana, Kenya, Rwanda, Tanzania and Uganda showed none of the surveyed hospitals had enough infrastructure to follow minimum standards and practices that the World Health Organization considers essential for provision of emergency and surgical care (66).

A systematic review of barriers to access and utilization of Emergency Obstetric Care (EmOC) in SSA found that barriers included lack of emergency obstetric care services and supplies, shortage of trained staff, poor management of emergency obstetric care provision, cost of services, long waiting times, poor referral practices, and poor coordination among staff (67). There are moreover stark inequities in the distribution of the little available. A systematic review of the geographical distribution of EmOC in sub-Saharan Africa showed a concentration of EmOC facilities in capitals, central and urban areas. At least a third of women in the sub-region cannot reach their nearest EmOC facility within the recommended two-hour timeframe (68). Sierra Leone's referral system is described as weak and vulnerable, with 75% of the country having insufficient access to essential health care (69).

Beyond SSA, similar challenges are recorded in some other low-and-lower-middle income countries (LMIC), and in some cases even upper middle and high-income countries – albeit of lower severity than in the LMIC context. Issues that have been found underlying the “why” and “how” of these challenges are similar to some of those we have found in this review of the “no bed syndrome” in Ghana such as human resources including overstretched, stressed and demotivated health workers, information and communication gaps, and inadequate equipment, tools, supplies, and infrastructure (7, 33, 70–73).

These multiple factors work as a tightly linked system rather than individually. “No bed syndrome” is a system problem. Unfortunately, the efforts that have been made to address the problem in Ghana to date appear to be piecemeal rather than an integrated and well synergized systems reform approach. A systems approach needs to address context, political and health system priorities, and values such as equity in access alongside addressing deficiencies in core health system functions or building blocks simultaneously. Addressing deficiencies in core health system functions or building blocks such as information systems and human resources will include agreeing on and adequately equipping public and private hospital emergency rooms with a basic minimum package of modern emergency care equipment to adequately manage emergencies according to international standards. It also requires the development and adequate funding of emergency and critical care training programs for health workers as sub-specialty training options as well as periodic in-service training updates for all frontline staff and managers of emergency rooms and care facilities. Experiences from other settings shows that legislation that imposes sanctions for refusal to offer first aid care to patients with life-threatening conditions before referring them if a facility cannot cope can make a difference (74). However, legislation will be ineffectual if not accompanied by an addressing of the resource challenges that make care unavailable.

Difficult as it is in the resource constrained context of a lower middle-income country like Ghana, more efforts need to be made to ensure adequate numbers of well trained and highly skilled emergency care staff are well distributed across the health system. This will require not only higher enrolment numbers but also better retention strategies such as better working conditions including higher salaries as well as infrastructure, tools and equipment and accessible career development opportunities. This can help to spread workload and reduce provider stress and burnout. It can also reduce referrals to the few relatively better-staffed facilities (in terms of skills and numbers as well as resources including equipment, tools, and supplies), and therefore reduce the likelihood of overwhelming the ability of bigger hospitals to handle emergencies.

The terminology “no bed syndrome” may not be in use outside Ghana but the problem of inadequate emergency health care access is a shared problem across many countries in Sub-Saharan Africa (SSA) and in low- and middle-income countries beyond SSA. This review from Ghana is therefore potentially valuable not only in pointing the way forward for change in Ghana but in other LMIC in SSA and beyond. It can contribute to moving global attention to emergency health care systems capacity and reform in low and middle income countries as part of the global SDG 3 sub-goal of UHC.

### Limitations of the study

This review was limited by the lack of research and peer reviewed publications on the specific terminology “no bed syndrome”. However, there was enough information from the grey and published literature and the wide media coverage of the issue in Ghana to provide clarity as to what “no bed syndrome” is and highlight its driving factors, and identify future research agendas that may solve the problem or encourage the monitoring and evaluation of the impact of solutions. Practically it was not possible to review the myriad of print and electronic media available in Ghana and the electronic and print media review focused only on the six high circulation media and the Ghana web. Though often the same story is carried by multiple media, it is possible we have missed some information.

## Conclusions

In conclusion:

(1) The “no bed syndrome” is a synonym for inadequate emergency health care access and reflects a neglect of emergency and critical health care in the UHC agenda.
(2) Integrated systems reform rather piecemeal and fragmented solutions are needed to address the problems.
(3) Though this review has focused on Ghana where the neglect of emergency and critical care has now become a problem attracting continuous public, media and legislator attention and scrutiny to the extent that a new terminology has become part of the popular discourse, the underlying problems would appear to be of concern well beyond Ghana.
(4) It is time to put emergency medical and surgical as well as obstetric care high on the UHC policy, implementation and research agenda for sub-Saharan Africa.
(5) Practically this should include:
  a. A systems approach that addresses the context, political and health system priorities, values such as equity in access alongside addressing deficiencies in core health system functions or building blocks simultaneously.
  b. Addressing deficiencies in core health system functions will include agreeing on and adequately equipping public and private hospital emergency rooms with a basic minimum package of modern emergency care equipment to adequately manage emergencies according to international standards.
  c. Development and adequate funding of emergency and critical care training programs for health workers as sub-specialty training options as well as periodic in-service training updates for all frontline staff and managers of emergency rooms and care facilities.
  d. Legislation that imposes sanctions for refusal to offer first aid care to patients with life-threatening conditions accompanied by an addressing of the resource challenges that make care unavailable.
  e. Ensuring adequate numbers of well trained and highly skilled emergency care staff are well distributed across the health system.

## Footnotes

^1^Ghana College of Physicians and Surgeons. Faculty of Public Health. Public health membership program. Cohort 13, Faculty of Public Health. Health Policy Brief: No Bed Syndrome in GHANA. Ghana College of Physicians and Surgeons. (Unpublished).
